# Synergistic Effects of Magnetic Z-Scheme g-C_3_N_4_/CoFe_2_O_4_ Nanofibres with Controllable Morphology on Photocatalytic Activity

**DOI:** 10.3390/nano13071142

**Published:** 2023-03-23

**Authors:** Yelin Chen, Ru Li, Lei Yang, Rongxu Wang, Zhi Li, Tong Li, Meijie Liu, Seeram Ramakrishna, Yunze Long

**Affiliations:** 1 Collaborative Innovation Center for Nanomaterials & Devices, College of Physics, Qingdao University, Qingdao 266071, China; 2 Instrumental Analysis Center of Qingdao University, Qingdao 266071, China; 3 Research Center for Intelligent & Wearable Technology, College of Textiles & Clothing, Qingdao University, Qingdao 266071, China; 4 Center for Nanofibers & Nanotechnology, Faculty of Engineering, National University of Singapore, Singapore 119077, Singapore; 5 State Key Laboratory of Bio-Fibers & Eco-Textiles, Qingdao University, Qingdao 266071, China

**Keywords:** magnetic, nanofibers, Z-scheme, photocatalyst

## Abstract

The rational design of interfacial contacts plays a decisive role in improving interfacial carrier transfer and separation in heterojunction photocatalysts. In Z-scheme photocatalysts, the recombination of photogenerated electron–hole pairs is prevented so that the redox capacity is maintained. Here, one-dimensional graphitic carbon nitride (g-C_3_N_4_)/CoFe_2_O_4_ fibres were synthesised as a new type of magnetic Z-scheme visible-light photocatalyst. Compared with pure g-C_3_N_4_ and CoFe_2_O_4_, the prepared composite photocatalysts showed considerably improved performance for the photooxidative degradation of tetracycline and methylene blue. In particular, the photodegradation efficiency of the g-C_3_N_4_/CoFe_2_O_4_ fibres for methylene blue was approximately two and seven times those of g-C_3_N_4_ and CoFe_2_O_4_, respectively. The formation mechanism of the Z-scheme heterojunctions in the g-C_3_N_4_/CoFe_2_O_4_ fibres was investigated using photocurrent spectroscopy and electrochemical impedance spectroscopy. We proposed that one of the reasons for the improved photodegradation performance is that the charge transport path in one-dimensional materials enables efficient photoelectron and hole transfer. Furthermore, the internal electric field of the prepared Z-scheme photocatalyst enhanced visible-light absorption, which provided a barrier for photoelectron–hole pair recombination.

## 1. Introduction

Environmental pollution and sustainable development of energy are among the most important issues in today’s society; research in these areas has, therefore, become increasingly focused on cost-effective and efficient solutions [1,2,3,4,5]. In particular, effective methods are required for the removal of organic pollutants that are rich in aromatic ring structures, such as antibiotics, as they are typically toxic, carcinogenic, and mutagenic. Tetracycline (TC) is commonly studied as a model contaminant because it is used extensively in livestock, fisheries, and human medicine and as a feed additive [6,7,8,9,10]. However, conventional degradation and biodegradation methods have limited efficiency in eliminating TC, thus resulting in residues in surface water, mud, and drinking water that pose a threat to both the natural environment and human health [11,12,13,14,15]. Pioneering work on anatase titanium dioxide (TiO_2_) semiconductors has provided a new avenue for addressing these challenges by employing photocatalysis [16,17,18]. However, TiO_2_ only absorbs ultraviolet (UV) light with a utilisation rate of only 2%; thus, new methods need to be developed to improve visible-light absorption rates [19,20].

Graphitic carbon nitride (g-C_3_N_4_), which is a non-metallic conjugated polymer, has recently attracted attention as a photocatalytic material owing to its satisfactory electronic energy band structure (forbidden band width of 2.7 eV) and physicochemical stability [21,22]. g-C_3_N_4_ has the advantages of being both inexpensive and easily available owing to the abundance of carbon- and nitrogen-containing raw materials in nature. However, g-C_3_N_4_ alone as a photocatalyst suffers from rapid charge recombination. To overcome this problem, g-C_3_N_4_ has been integrated with various materials, including metal co-catalysts [23], metal oxides [24], chalcogenides [25], metal–sulfur compounds [26], and spinel ferrites [27]. CoFe_2_O_4_, a typical bimetallic spinel ferrite, is characterised by its high stability and low cost. Moreover, CoFe_2_O_4_ is more biocompatible than monophase cobalt catalysts because the presence of Fe^3+^ reduces Co^2+^ spillover, thus inhibiting toxicity and secondary contamination arising from cobalt ion leaching from the material surface. Although CoFe_2_O_4_/g-C_3_N_4_ binary nanomaterials have been reported for visible photocatalytic pollutant degradation, these materials have been applied in block (three-dimensional) or sheet (two-dimensional) forms. In contrast, one-dimensional fibrous structures have rarely been investigated [28]. One-dimensional fibres can limit charge transfer in space, thus improving the transport efficiency of photogenerated electrons and holes and providing large surface areas and high porosity. This increases the number of active sites for the catalytic reaction and promotes photocatalytic activity.

Conventional heterojunctions, in which electrons are separated from holes by charge transfer, often have low redox potentials because active sites with high redox potentials are not retained [29]. In contrast, Z-scheme heterojunctions are depleted because of internal interactions between the materials, which result in the complexation of a specific fraction of electrons and holes, thus preserving the active sites with the highest redox potentials [30,31]. Zhang et al. used mixing and programmed heating to synthesise Z-scheme KBiO_3_/g-C_3_N_4_ heterojunction photocatalysts, which exhibited considerably improved photooxidative activity for the degradation of crystalline violet and phenol. Javad et al. designed and demonstrated a Z-scheme g-C_3_N_4_/BiVO_4_ heterostructure, which achieved a four-fold improvement in performance compared with pure BiVO_4_.

**Scheme 1:**
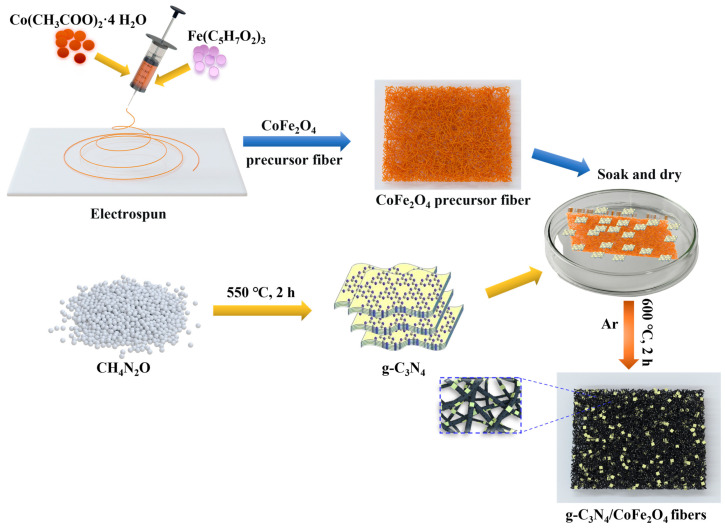
Synthesis of the composite photocatalysts used in this study.

Inspired by these findings, we synthesised a series of one-dimensional Z-scheme g-C_3_N_4_/CoFe_2_O_4_ heterojunction catalysts using an electrostatic spinning technique combined with high-temperature calcination (Scheme 1). The prepared catalysts were found to have good physicochemical properties, including increased surface area, enhanced visible-light absorption, and high recyclability. These photocatalysts were shown to effectively enhance electron transfer efficiency and concomitantly prolong the involvement of photogenerated carriers over the degradation period. In addition, the charge transfer mechanism of the synthesised Z-scheme heterojunction structure was proposed. The active substances that play a leading role in the removal of organic pollutants were identified by conducting quenching experiments and electron paramagnetic resonance (EPR) spectroscopy.

## 2. Experimental

### 2.1. Synthesis of the One-Dimensional Z-Scheme g-C_3_N_4_/CoFe_2_O_4_ Heterojunction Catalysts

The g-C_3_N_4_/CoFe_2_O_4_ binary catalysts were prepared using electrostatic spinning and high-temperature calcination. First, pure g-C_3_N_4_ and CoFe_2_O_4_ (denoted as CN and CFO, respectively) were prepared as described in the Supporting Information. Then, CoFe_2_O_4_ was mixed with dried electrostatically spun g-C_3_N_4_ fibre films to the desired mass ratio (0.01, 0.05, 0.1, 0.15, or 0.2 g-C_3_N_4_: CoFe_2_O_4_), soaked in a cuvette for 3 h, and dried at 60 °C. The one-dimensional g-C_3_N_4_/CoFe_2_O_4_ nanofibre films were then prepared by placing the dried mixture in a tube furnace under an argon atmosphere and ramping the temperature to 600 °C (1 °C/min) for 2 h. The products were denoted as CN/CFO-0.01, CN/CFO-0.05, CN/CFO-0.1, CN/CFO-0.15, and CN/CFO-0.2 based on the g-C_3_N_4_ mass ratio.

### 2.2. Photocatalytic Degradation

#### 2.2.1. Photocatalytic Degradation Experiments

To study its photocatalytic degradation activity, the selected catalyst (25 mg) was placed in a quartz glass tube containing 50 mL of contaminant solution (TC 10 mg L^−1^) or methylene blue (MB, 50 mg L^−1^). The mixture was then subjected to a dark reaction for 60 min to bring the catalyst to a stable adsorption state. The system was then irradiated with a Xenon lamp (300 W) with a 420 nm cut-off filter to initiate the photodegradation reaction. Every 30 min, 3 mL of the solution was removed with a 5 mL pipette for concentration measurements.

#### 2.2.2. Free Radical Capture Experiments

To determine the main active components of the catalytic process, free radical scavengers were used before the start of the reaction. 4-Hydroxy-2,2,6,6-tetramethyl-1-piperidinyloxy (TEMPOL, 10 mM) was used to scavenge superoxide radicals (·O^2−^), ammonium oxalate (AO, 10 mM) to scavenge photogenerated holes (h^+^), isopropyl alcohol (IPA, 10 mM) to scavenge hydroxyl radicals (·OH), and silver nitrate (AgNO_3_, 10 mM) to scavenge electrons (e^−^) [32].

## 3. Results and Discussion

### 3.1. Structure and Morphology of CN/CFO

**Figure 1:**
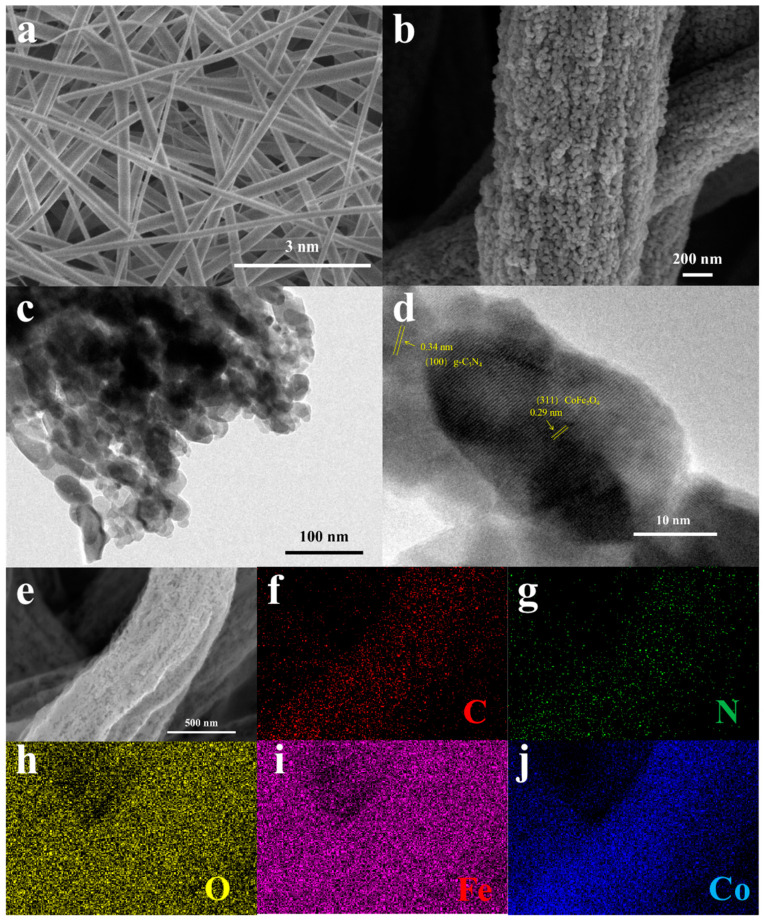
SEM images of CN/CFO-0.05 (**a**,**b**,**e**); TEM images of CN/CFO-0.05 (**c**,**d**); EDS elemental mapping images of CN/CFO-0.05 (**f**–**j**).

**Figure 2:**
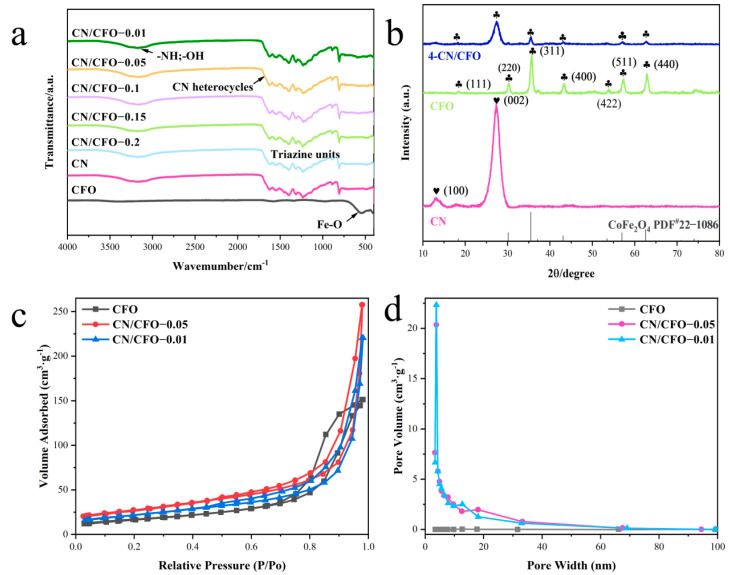
FT-IR patterns of pure CN, CFO and composite samples of different proportions (**a**); X-ray diffraction patterns of pure CN, CFO, and CN/CFO-0.05 (**b**); N_2_ adsorption–desorption isotherms of pure CFO, CN/CFO-0.05, and CN/CFO-0.01 (**c**); pore size distribution of pure CFO, CN/CFO-0.05, and CN/CFO-0.01 (**d**).

Scanning electron microscopy (SEM) images of the calcined CN/CFO composite fibre films are shown in Figure 1a, with the corresponding transmission electron microscopy (TEM) images shown in Figure 1b. These images depict that the intricate fibrous morphology of the CN/CFO-0.05 composite fibrous film consists of lamellar g-C_3_N_4_ with granular CoFe_2_O_4_ uniformly distributed through the nanofibres. The fibres, which had diameters of 300–400 nm, formed a rough mesh structure. Such a microstructure greatly improves the transport efficiency in solution, increases the amount of active reaction sites, and improves the stability of the catalyst. As shown in Figure 1d, the observed lattice widths of 0.25 and 0.48 nm correspond to the crystal planes of CoFe_2_O_4_ (311) and (111), respectively, in agreement with the obtained X-ray diffraction (XRD) results (Figure 2b). Energy-dispersive spectroscopy (EDS) element mapping (Figure 1f–j) also reveal that the elements are evenly distributed, indicating the successful formation of a composite between the two catalytic materials.

The chemical structure and bonding of the composite fibre films were studied by Fourier transform infrared spectroscopy (FT-IR) (Figure 2a). All the composite catalysts exhibit characteristic absorption bands at approximately 1628, 1538, 1394, 1318, and 1240 cm^−1^, which correspond to the stretching vibrations of the triangular C-N(-C)-C and C-NH-C units in the C-N heterocyclic system. The absorption band at approximately 801 cm^−1^ can be attributed to the breathing mode vibration of the triazine unit, and the band at approximately 572 cm^−1^ is derived from Fe–O bonding at the octahedral site of the spinel CoFe_2_O_4_ unit. The characteristic absorption bands of both CFO and CN correspond to those observed in the spectra of the various CN/CFO photocatalysts, indicating successful composite formation. In addition, the broad bands at 3000–3500 cm^−1^ may be attributed to the stretching vibrations of hydroxyl (–OH) and hydrogen bonds resulting from the adsorption of water from the environment [33].

The effect of composite formation with CN on the structure of CFO was investigated using XRD (Figure 2b). Here, crystal diffraction peaks corresponding to g-C_3_N_4_ (100) and (002) are visible at 13.0° and 27.4°, respectively [34]. The (100) and (002) crystal planes are formed by interlayer stacking of the repeating structure of the tri-s-triazine unit and the laminar structure of the conjugated aromatic ring, respectively. Conversely, CFO exhibited characteristic diffraction peaks at 18.2°, 35.5°, 43.5°, 57.2°, and 62.7°, corresponding to the (111), (311), (400), (511), and (440) crystal faces of spinel CoFe_2_O_4_, respectively (PDF card no. 22-1086) [35]. The composite catalysts exhibited the characteristic peaks of both CN and CFO to higher degrees, indicating that the material is relatively crystalline. These results also indicate that composite formation has little effect on the lattice structure.

Figure 2c displays the N_2_ adsorption–desorption isotherms of CFO, CN/CFO-0.05, and CN/CFO-0.01. Each isotherm exhibits an adsorption hysteresis loop, which corresponds to a system in which capillary coalescence of the porous adsorbent occurs. These features are consistent with those of type IV isotherms, according to the latest IUPAC classification, and are characteristic of mesoporous materials [36]. As shown in Figure 2d, the catalyst materials exhibit pore size distributions of 4–15 nm. The similar porous structure provides more active sites for the adsorption process, thus enabling the enhancement of photocatalytic performance in this material.

Table 1 shows the specific surface area values of CFO, CN/Cfos-0.05 and CN/Cfos-0.01. CN/Cfos-0.05 has the largest specific surface area and provides the most reaction sites for the redox reaction in the photocatalytic process.

**Figure 3:**
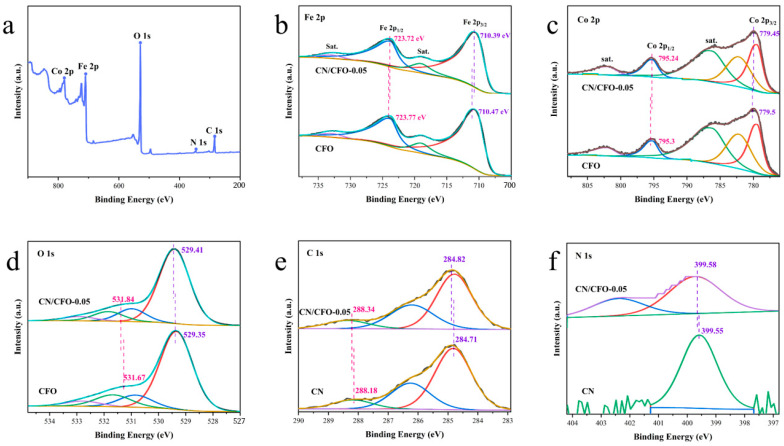
Measured XPS spectrum of CN/CFO-0.05 (**a**); narrow-band Co 2p, Fe 2p, O 1s, C 1s, and N 1s spectra of CFO, CN, and the CN/CFO composites (**b**–**f**).

Figure 3a shows the atomic composition of CN/CFO-0.05 as determined by X-ray photoelectron spectroscopy (XPS). Here, peaks corresponding to Co 2p, Fe 2p, O 1s, C 1s, and N 1s are observed, indicating the successful preparation of the Z-scheme heterojunctions. Figure 3b–f shows the high-resolution Co 2p, Fe 2p, O 1s, C 1s, and N 1s spectra of the catalysts. The observed peak values in the Fe 2p spectrum are 710.47 and 723.77 eV, respectively, which can be attributed to Fe 2p_1/2_ and Fe 2p_3/2_, both of which are derived from Fe^3+^. These peaks were shifted towards lower binding energies by 0.05 and 0.08 eV, separately, for the CN/CFO composites (Figure 3b). Figure 3c further shows that because of the characteristics of the CN/CFO composites, the Co 2p_1/2_ and Co 2p_3/2_ spectral peaks of CFO (779.5 and 795.3 eV, respectively) also shift by 0.06 and 0.05 eV, respectively. In the fitted O 1s spectrum of CFO (Figure 3d), the observed lattice oxygen (529.41 eV) and hydroxyl-oxygen (531.84 eV) peaks indicate the adsorption of oxygen species onto the material surface. In the C 1s spectra (Figure 3e), the peaks of 284.82 and 288.18 eV are shifted towards higher binding energies in the composite by more than 0.1 eV because of the strong interactions between the CN and CFO components. The characteristic N 1s peak, which was attributed to C–N bonds, was shifted towards higher binding energies by 0.03 eV for the CN/CFO composites (Figure 3f). This higher binding energy arises because the electrons in the CN conduction band (CB) form complexes with the holes in the CFO valence band (VB) [37]. High performance Z-scheme photocatalysts can be prepared by edge selective deposition coupled with long terminal junctions and interfacial heterojunctions [37].

### 3.2. Photocatalytic and Photovoltaic Properties of CN/CFO

**Figure 4:**
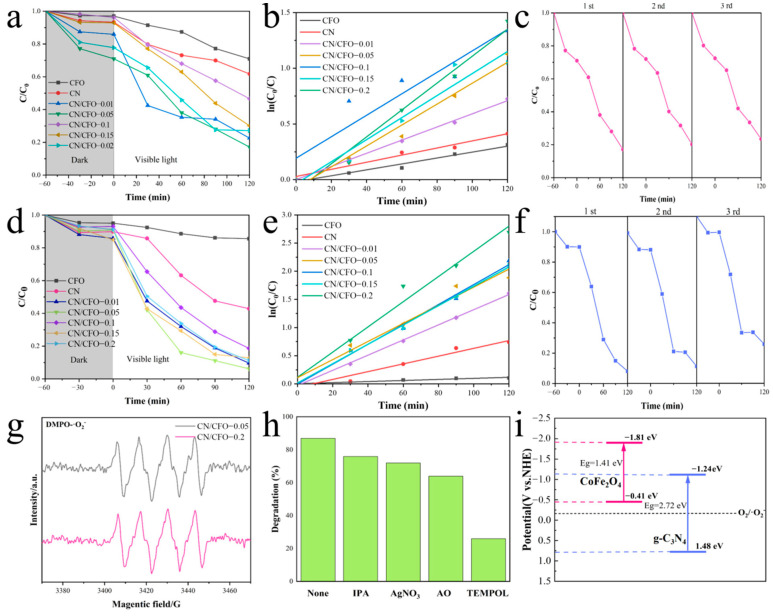
Photocatalytic degradation efficiency of CN/CFO for TC, first-order reaction kinetics, and degradation efficiency after three cycles (**a**–**c**); photocatalytic degradation efficiency of CN/CFO for MB, first-order reaction kinetics, and degradation efficiency after three cycles (**d**–**f**); EPR spectra of CN/CFO-0.05 and CN/CFO-0.01 in the presence of DMPO (**g**); radical capture experiment results (**h**); energy band structure of the catalysts (**i**).

The efficiency of photocatalysed pollutant degradation of different compositions of CN/CFO was investigated to determine the optimal material ratio. Photocatalytic degradation experiments were, therefore, carried out using TC and MB as the model pollutants (Figure 4a,d). To obtain absorption equilibrium, the catalyst/pollutant systems were placed in darkness for 60 min at the first stage of each reaction. The observed TC degradation rate of CN/CFO-0.05 was approximately 3 and 2.3 times that of CFO and CN, respectively, indicating that the Z-scheme heterojunction composite membrane provides a considerable improvement in photocatalytic efficiency. The degradation experiment with MB showed a similar trend, giving a maximum degradation efficiency of 95%.

For the degradation of TC and MB, the CN/CFO-0.05 photocatalyst exhibited the largest promotion effect (Figure 4b,e). The photocatalytic reaction kinetic data was fitted to a Langmuir–Hinshelwood kinetic model (ln(*C*_0_/*C*) = *kt*, where *C*_0_ is the pollutant concentration before the reaction was initiated and *C_t_* is the corresponding concentration at *t* min during the photocatalytic reaction process). CN/CFO-0.05 exhibited the highest catalytic activity for TC degradation with a reaction rate constant of 0.01207 min^−1^, which was 4.6 and 3.8 times higher than those of CFO (0.00265 min^−1^) and CN (0.00319 min^−1^), respectively. The same trend was observed for the degradation of MB. This substantially enhanced catalytic performance of the composite fibrous membranes may, therefore, indicate the inhibition of photoelectron–hole pair recombination caused by the Z-scheme heterojunction.

The recoverability and recyclability of the photocatalysts were also investigated (Figure 4c,f). After three cycles, the catalytic efficiency of the CN/CFO composite fibrous membrane for TC degradation decreased by only 6%, thus indicating that the one-dimensional Z-scheme CN/CFO heterojunction structure has good chemical stability.

EPR experiments using 5,5-dimethyl-1-pyrroline *N*-oxide (DMPO) as a free radical trapping agent were performed to reveal the mechanism of TC photocatalytic degradation. Figure 4g shows that the high-intensity DMPO–O^2−^ signal, which cannot be detected in dark conditions, is observed under visible light. This indicates that superoxide radicals (·O^2−^) play a crucial role in the photocatalytic degradation mechanism [38].

Photocatalytic radical trapping experiments were also performed by adding free radical trapping agents in the presence of the catalyst (Figure 4h). All the trapping agents inhibit the degradation of TC, suggesting that the presence of oxides promotes the degradation mechanism. In particular, a high degree of inhibition is observed for TC degradation following the addition of TEMPOL as a superoxide radical (·O^2−^) trapping agent [39].

The contribution of superoxide radicals in the catalytic process of CN/CFO is dominant, as confirmed by the EPR and free radical trapping experiments. Subsequently, the energy band structures of the catalysts were plotted using the obtained XPS VB data (see Section 3.3) and the measured bandgap (*E*_g_) of the catalysts, as shown in Figure 4i. The obtained CB potentials were −1.24 and −1.81 eV (vs. NHE) for CN and CFO, respectively, whereas the VB potentials were 1.48 and −0.41 eV (vs. NHE) for CN and CFO, respectively.

**Figure 5:**
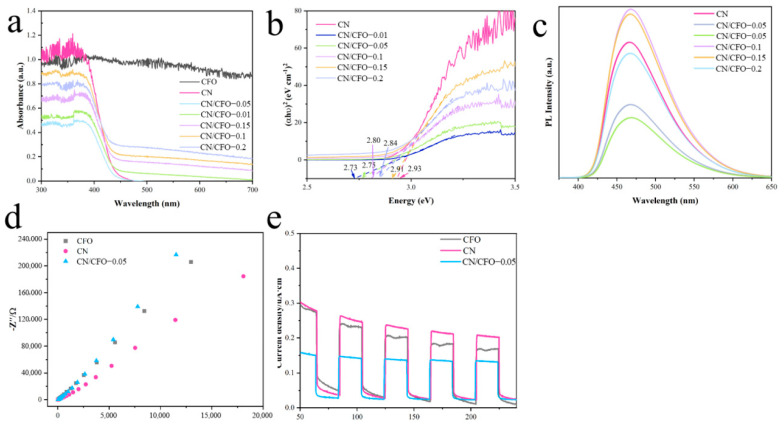
UV–Vis absorption spectra (**a**), band gaps (**b**), and PL spectra (**c**) of CFO, CN, and CN/CFO-0.05; EIS impedance profiles of CFO, CN, and CN/CFO-0.05 (**d**); transient photocurrent responses of CFO, CN, and CN/CFO-0.05 (**e**).

The light absorption capacities of the composite fibre films were evaluated by ultraviolet–visible (UV–Vis) absorption spectroscopy (Figure 5a). CN, which is responsive to UV and visible light, exhibits an absorption edge of approximately 440 nm, which corresponds to a bandgap of 2.93 eV (Figure 5b). Conversely, CN/CFO exhibited lower-wavelength absorption edges with larger absorbances of the visible range. This indicates that the addition of CN broadens the absorption range of CFO in visible light. The red shift in the absorption band of the CN/CFO composite may result from the composition of the Z-scheme heterojunction, which allows the effective separation of electron–hole pairs, thus maintaining a high redox potential and increasing photocatalytic activity.

The *E*_g_ values of the photocatalysts were calculated using Equation (1):

*αhν* = A(*hν* − *E*_g_)^n/2^ (1)

where *α* is the absorption coefficient, *h* is the Planck’s constant, *ν* is the optical frequency, *A* is the constant, and *E*_g_ is the bandgap energy [40]. *n* is related to the optical transition type of the semiconductor (direct-transition semiconductor, *n* = 1; indirect-transition semiconductor, *n* = 4). CFO and CN are direct-transition semiconductors [41]; thus, the band gaps of the hybrid fibrous films, CFO, and CN were obtained by plotting (*αhν*)^2^ vs. *hν* (Figure 5 band listed in Table 2). The calculated band gap values for CN, CN/CFO-0.01, CN/CFO-0.05, CN/CFO-0.1, CN/CFO-0.15, and CN/CFO-0.2 were 2.73, 2.75, 2.80, 2.84, 2.91, and 2.93 eV, respectively. A smaller bandgap indicates a larger visible-light harvesting capability and, more importantly, improved photocatalytic performance in the visible region.

To investigate the separation efficiency of the photogenerated electrons (e^−^) and holes (h^+^), photoluminescence (PL) spectra of the photocatalysts were obtained (Figure 5c). Because the PL emission peaks are caused by photogenerated electrons and holes, the relatively low emission peak intensity corresponds to a reduced carrier complexation efficiency [42]. CN/CF-0.05, which exhibits the largest photocatalytic activity, has the smallest PL spectral intensity, which is consistent with the results of previous photocatalytic studies.

The electron-transfer efficiencies and pathways of the catalysts were further assessed using electrochemical impedance spectroscopy (EIS). In general, the smaller the radius of the arc in the electrochemical impedance spectrum, the smaller is the charge-transfer resistance. Figure 5d shows that CN/CFO-0.05 exhibits the smallest radius, indicating that this catalyst has the largest electron-transfer efficiency [43]. Thus, the CN/CFO Z-scheme heterojunction structure enhanced the conductivity and mobility of the photocatalyst and contributed to the improved photocatalytic performance, as shown by the PL and UV–Vis results.

Transient photocurrent response (*I*–*t*) experiments were then performed to investigate the photogenerated carrier separation efficiency and current density properties of the catalysts [44]. As shown in Figure 5e, CN/CFO-0.05 exhibits a higher photocurrent density than the other samples under cyclic light switching conditions. This behaviour may be attributed to the suppression of photogenerated carrier recombination by the Z-scheme heterojunction, which results in a higher visible-light absorption capacity, electron transport capacity, and electron–hole separation efficiency, thus leading to enhanced photocatalytic activity. In addition, the *I*–*t* curve for CN/CFO-0.05 remained constant after five on/off cycles, which demonstrates the good electrochemical stability of the catalyst.

The degradation efficiencies of various previously reported catalysts for TC degradation were compared with those of the catalysts in the present study, as shown in Table 3. The one-dimensional Z-scheme heterojunction photocatalyst prepared in this study showed improved catalytic efficiency than other types of photocatalysts for the degradation of TC at the same concentration under visible-light irradiation.

### 3.3. Determination of the Photocatalytic Mechanism

**Figure 6:**
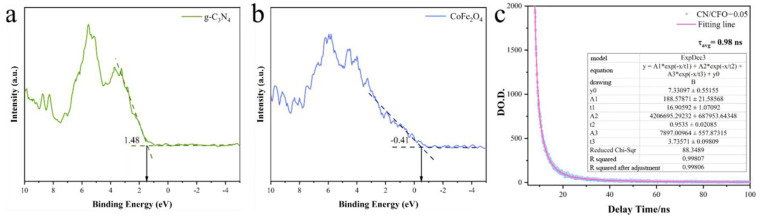
Valence band positions of CN, CFO (**a**,**b**); transient fluorescence lifetime of CN/CFO-0.05 (**c**).

The valence band energies (*E*_VB_) of CFO and CN were determined using XPS VB spectroscopy (Figure 6a,b). The *E*_VB_ values of CFO and CN were −0.41 and −1.48 eV, respectively. The *E*_g_ values for CFO and CN were 1.4 and 2.72 eV, as calculated from Figure 5b. The conduction band energies (*E*_CB_) of CFO and CN were then calculated using Equation (2) [45]:

*E*_VB_ = *E*_CB_ + *E*_g_ (2)

The calculated ECB values for CFO and CN were −1.81 and −1.24 eV, respectively.

To understand the energy relaxation and recombination processes of photogenerated carriers in the photocatalysts, their kinetic decays were investigated using transient fluorescence lifetime measurements (Figure 6c). Fitting revealed that a ternary exponential function best described the carrier decay kinetics [13], as follows:

*y* = *y*_0_ + *A*_1_e^−*x*/*τ*1^ + *A*_2_e^−*x*/*τ*2^ + *A*_3_e^−*x*/*τ*3^ (3)

where *y* is the PL intensity at a given time; *A*_1_, *A*_2_, and *A*_3_ are the weighting factors of the different time components; and *τ*_1_, *τ*_2_, and *τ*_3_ are the time decay constants of the different time components. The weighted average lifetime of the fitted data (*τ*_avg_ = 0.98 ns) was calculated using Equation (4):

*τ*_avg_ = (*A*_1_*τ*_1_^2^ + *A*_2_*τ*_2_^2^ + *A*_3_*τ*_3_^2^)/(*A*_1_*τ*_1_ + *A*_2_*τ*_2_ + *A*_3_*τ*_3_) (4)

The smaller time constants *(τ*_2_ and *τ*_3_*)* are attributed to the elimination of excited emission and the relaxation process associated with carrier recombination in the electric field within the Z-scheme heterojunction. The larger time constant (*τ*_1_) is attributed to the complexation process between carriers involving the surface states, where a longer carrier lifetime leads to higher photocatalytic activity [46].

**Figure 7:**
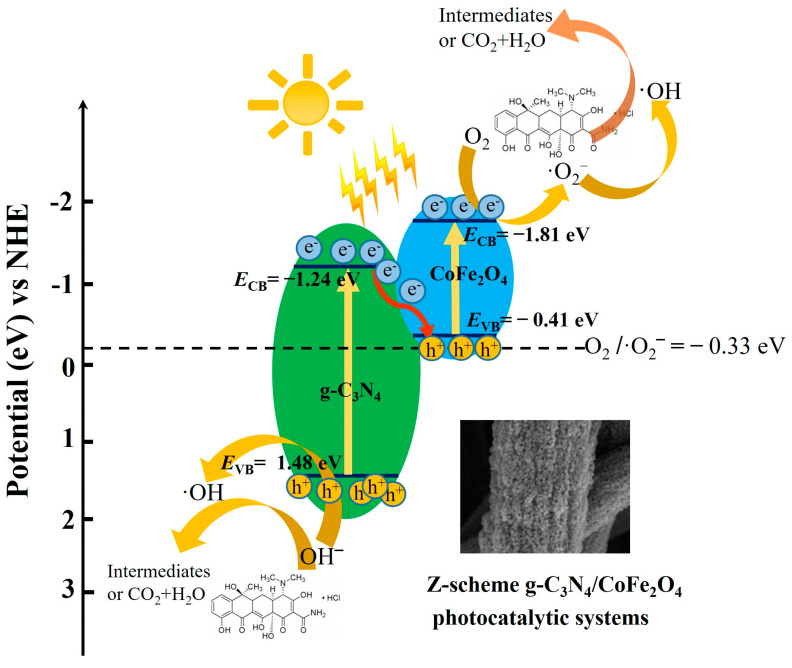
Electron-transfer pathways in the Z-scheme CN/CFO heterojunction composite photocatalysts.

A possible electron-transfer pathway for the Z-scheme CN/CFO heterojunction composite photocatalyst is shown in Figure 7. The photogenerated electrons in the g-C_3_N_4_ CB are complexed with holes in the CoFe_2_O_4_ VB, which results in the in situ accumulation of electrons in the CoFe_2_O_4_ CB and holes in the g-C_3_N_4_ VB, thus preserving the optimum redox potential. This effectively separates the electrons and holes and deposits them in the g-C_3_N_4_ VB and CoFe_2_O_4_ CB, respectively [47]. The pathway for the photodegradation of TC and MB by the catalyst can be expressed by the following equations:

CN/CFO + hυ → CFO(e^−^) + CN(h^+^) (5)

CFO (e^−^) + O_2_ →·O^2−^ (6)

O^2−^ + TC/MB → Intermediates + CO_2_ + H_2_O (7)

h^+^ + H_2_O→ (•OH) (8)

•OH + TC/MB → Intermediates + CO_2_ + H_2_O (9)

h^+^ + TC/MB → Intermediates + CO_2_ + H_2_O (10)

Based these findings, a model of the Z-scheme heterojunction catalyst constructed from CN and CFO is proposed, as shown in Figure 7. The photogenerated electrons (e^−^) are transferred from the g-C_3_N_4_ CB position to the CoFe_2_O_4_ VB position, where they combine with the photogenerated holes (h^+^), thereby increasing the number of electrons and holes. For the degradation of TC or MB, the CB reacts with dissolved O_2_ to form ·O^2−^. The photogenerated pores in g-C_3_N_4_ VB can then directly participate in the degradation reaction or form hydroxyl radicals (·OH) from H_2_O, leading to the degradation of TC or MB.

## 4. Conclusions

In this study, a type of one-dimensional g-C_3_N_4_/CoFe_2_O_4_ Z-scheme heterojunction photocatalyst was prepared by electrostatic spinning combined with high-temperature calcination. The Z-scheme g-C_3_N_4_/CoFe_2_O_4_ composites exhibited good physicochemical properties, including high specific surface areas, visible light absorption, and recyclability. Subsequently, the prepared composites exhibited clear photocatalytic activity for TC and MB degradation: in particular, the degradation efficiency of the g-C_3_N_4_/CoFe_2_O_4_-0.05 catalyst for TC degradation was increased 4.6- and 3.8-fold compared with those of CoFe_2_O_4_ and g-C_3_N_4_, respectively. Additionally, this catalyst achieved the highest photocatalytic efficiency of the composites synthesised in this study. Furthermore, the catalytic efficiency of g-C_3_N_4_/CoFe_2_O_4_-0.05 for MB degradation was 3.3 and 2.4 times larger than those of CoFe_2_O_4_ and g-C_3_N_4_, respectively. The dominant contribution of superoxide radicals in this process was confirmed by quenching experiments and EPR spectroscopy. Finally, on the basis of the experimental results and theoretical calculations, the core mechanism of charge transfer in the Z-scheme heterojunction structures was proposed.

## Figures and Tables

**Table 1:** The specific surface area of the catalyst.

Materials	BET (m^2^/g)
CFO	60.261
CN/CFO-0.01	77.694
CN/CFO-0.05	97.012

**Table 2:** Band gap of catalyst.

Materials	E_g_ (eV)
CN	2.93
CN/CFO-0.01	2.73
CN/CFO-0.05	2.75
CN/CFO-0.1	2.80
CN/CFO-0.15	2.84
CN/CFO-0.2	2.91

**Table 3:** Comparison of TC removal rates by different photocatalysts under 300 W xenon lamp irradiation.

Material	Light Source	Photocatalyst Dosage (g/L)	Initial Concentration of TC (mg L^−1^)	Degradation Rate (%)	Ref.
g-C_3_N_4_/CoFe_2_O_4_	300 W Xe lamp	0.2	20	84 (120 min)	This work
ZnMnO_4_/BiOI	300 W Xe lamp	0.4	20	87.2 (80 min)	[23]
BiVO_4_/BiOI	300 W Xe lamp	0.5	20	69.57 (60 min)	[35]
La_2_O_3_/g-C_3_N_4_	300 W Xe lamp	2	20	93 (120 min)	[38]
C_3_N_4_/CoFe_2_O_4_ (bulk)	300 W Xe lamp	0.5	20	85.2 (240 min)	[43]
BiOI/g-C_3_N_4_/CoFe_2_O_4_	300 W Xe lamp	0.6	20	87.3 (180 min)	[45]

## Supplementary Materials

The following supporting information can be downloaded at: https://www.mdpi.com/article/10.3390/nano13071142/s1. References [48,49] are cited in the Supplementary Materials.
