# Supplementary material for: Synergistic Effects of Magnetic Z-Scheme g-C3N4/CoFe2O4 Nanofibres with Controllable Morphology on Photocatalytic Activity
Source: Nanomaterials (Basel). 2023 Mar 23;13(7):1142. doi: 10.3390/nano13071142 (PMC10096916; doi:10.3390/nano13071142)
Supplement: Supplementary file 1 [file nanomaterials-13-01142-s001.zip › nanomaterials-2290808-supplementary.pdf]

Supporting Information

Controllable morphology magnetic g-C_3N_4/$CoFe_2O_4$ nanofibers photocatalysts with Z-scheme enhance photocatalytic performance

EXPERIMENTAL SECTION

Materials: Polyvinylidene fluoride (PVDF, Mw & 550000) was obtained from Sigma-Aldrich (St. Louis, MO). Acetone (CH_3COCH_3), N-imethylformamide (DMF), methylene blue (MB), cobalt acetate tetrahydrate ($Co(CH_3COO)_2{\cdot}4H_2O$), ferric acetylacetonate ($Fe(C_5H_7O_2)_3$), Urea, silver nitrate ($AgNO_3$), ammonium oxalate (AO), 4-hydroxy-2,2,6,6-tetramethylpiperidoxy (TEMPOL) and isopropanol (IPA) were purchased from Sinopharm Chemical Reagents Co, LTD. Tetracycline (TC) was provided by Shanghai Maclin Biochemical Co, LTD. These chemicals were used in all experiments with analytic grade, deionized and highly purified water.

Characterization

X-ray diffraction (XRD) data were obtained on a Rigaku Smart Lab X-ray diffractometer with Cu Kα radiation (0.1542 nm) using a step size of 0.02°. We observed the morphology of the samples using transmission electron microscopy (TEM, JEM-2100F) equipped with an energy-dispersive X-ray spectrometer (EDS, INCAx-Sight6427). Flourier transformed infrared spectroscopic (FTIR) spectra were obtained on a Nicolet iS50 spectrometer. The photoluminescence (PL) spectrum of the sample at the excitation wavelength of 320 nm was measured by the Hitachi F-4600 fluorescence spectrometer to study the recombination efficiency of a photoinduced charge.

UV-visible diffuse reflection spectra (UV–Vis DRS) were recorded on a SOLID 3700 spectrophotometer. We utilized X-ray photoelectron spectroscopy (XPS, Thermo ESCALAB 250) signals to evaluate the samples' elements with an Al Ka X-ray source ($h\nu$ = 1486.6 eV) and pass energy of 30 eV. The Brunauer-Emmett-Teller specific surface area of the sample was measured on a Quantachrome Autosorb-IQ-MP/XR nitrogen adsorption device. Photoelectrochemical measurement was performed by electrochemical workstation (CH Instruments CHI 660E scanning potentiostat). Three electrodes with sample, Pt electrode, and saturated calomel electrode were placed in the electrolyte of 0.5 $mol \cdot L^{-1}$ Na_2SO_4 aqueous solution. The hybrid fiber membranes were cut into units of 1 cm × 1 cm and fixed on an indium tin oxide glass by conductive adhesive. Photocurrent density data were recorded every 20 s under an Xe lamp (300 W) with a UV cut filter (λ > 420 nm). The degradation intermediates of TC were identified by Ultimate 3000 UHPLC-Q precision liquid chromatography-mass spectrometry (LC-MS).

Preparation of g-C_3N_4 nanosheets

g-C_3N_4 was prepared by calcination. A quantity of urea was placed in a crucible and sent to a muffle furnace at a heating rate of 5 °C/min to 550 °C and maintained for 2 h. The product was labeled as CN [1].

Preparation of $CoFe_2O_4$ precursor fibers

$CoFe_2O_4$ precursor fibers were prepared by electrostatic spinning method. 2 g acetone (CH_3COCH_3) and 2 g DMF (N-N dimethylformamide), 0.8 g PVDF were mixed (water bath with magnetic stirring heated at 50 °C for ~2-3 h); 1 mmoL (0.249 g) of cobalt $Co(CH_3COO)_2$-4 H_2O acetate tetrahydrate and 2 mmoL (0.706 g) of iron acetylacetonate $Fe(C_5H_7O_2)_3$ were dissolved into the just mixed solution; then the mixture required for electrostatic spinning was obtained after

continued stirring in a 40 °C water bath until overnight (about 15 h) [2]. Electrostatic spinning is then performed with a propulsion speed of 16 μL, a voltage of 15 kV, and an acceptance distance of 15 cm. The product was then dried in an oven under vacuum at 70 °C for 12 h. The product was labeled CFO.

References:

1. Ye, J.; Dai, J.; Yang, D.; Li, C.; Yan, Y.; Wang, Y. 2D/2D confinement graphene-supported bimetallic Sulfides/g-C_3N_4 composites with abundant sulfur vacancies as highly active catalytic self-cleaning membranes for organic contaminants degradation. *Chemical Engineering Journal* **2021**, *418*, 129383.[CrpssRef]
2. Paul, A.; Dhar, S.S. Designing $Cu_2V_2O_7/CoFe_2O_4$/g-C_3N_4 ternary nanocomposite: A high performance magnetically recyclable photocatalyst in the reduction of 4-nitrophenol to 4-aminophenol. *Journal of Solid State Chemistry* **2020**, *290*, [CrpssRef]
